# A predictive model to assess the risk of developing hyperlipidemia in patients with type 2 diabetes

**DOI:** 10.1371/journal.pone.0315781

**Published:** 2025-02-14

**Authors:** Rujian Ye, Xitong Huang, Hehui Yang, Wei Pan, Ping Wang, Janhao Men, Dawei Huang, Shan Wu

**Affiliations:** 1 The People’s Hospital of Yuhuan, Yuhuan, Zhejiang, China; 2 Tianjin Dongli District Hospital of Traditional Chinese Medicine, Tianjin, China; University of Montenegro-Faculty of Medicine, MONTENEGRO

## Abstract

**Background:**

Type 2 diabetes (T2D) is increasingly recognized as a significant global health challenge, with a rising prevalence of hyperlipidemia among diabetic patients. Effectively predicting and reducing the risk of hyperlipidemia in T2D patients to mitigate their cardiovascular risk remains an urgent issue.

**Objectives:**

The research sought to determine early clinical indicators that could predict the onset of hyperlipidemia in patients with T2D and to establish a predictive model that integrates clinical and laboratory indicators.

**Methods:**

A cohort of T2D patients, excluding those with pre-existing hyperlipidemia or confounding factors, was analyzed. Clinical and laboratory data were used in a LASSO regression model to select key predictive variables. A nomogram was then constructed and evaluated using receiver operating characteristic (ROC) analysis and calibration.

**Results:**

Among 269 participants, PCSK9 levels were significantly elevated in T2D patients with hyperlipidemia and exhibited a positive correlation with several lipid markers. LASSO regression identified six predictors: BMI, TG, TC, LDL-C, HbA1c, and PCSK9. The nomogram model exhibited robust predictive performance (AUC, 0.89 (95% CI: 0.802–0.977)) and showed good calibration.

**Conclusions:**

This method effectively predicts the risk of hyperlipidemia in patients with T2D and provides a valuable tool for early intervention. PCSK9, as a key predictor, highlights its potential role in the pathogenesis of diabetes with hyperlipidemia and offers new avenues for targeted therapy.

## Introduction

Diabetes, particularly type 2 diabetes (T2D), is rapidly becoming a global health concern. An analysis of high-quality data from 138 countries predicted that the global prevalence of diabetes will rise to 10.2% by 2030 and reach 10.9% by 2045 [1]. Among the elderly population aged 65-99 years, the prevalence of diabetes is expected to climb to 19.3% by 2045 [2].

Owing to factors such as insulin resistance, chronic hyperglycemia, and abnormal lipid metabolism, patients with T2D are particularly prone to developing hyperlipidemia [3]. Insulin resistance is closely linked to elevated triglyceride (TG) levels and reduced high-density lipoprotein cholesterol (HDL-C) levels [4]. In developing countries such as Jordan, 91.4% of T2D patients exhibit hyperlipidemia, with the most common pattern being low HDL-C and high LDL-C [5]. Studies conducted in India have shown that mixed hyperlipidemia is most prevalent among T2D patients, particularly those with a higher atherogenic plasma index [6]. Insulin resistance not only contributes to hyperlipidemia in diabetic patients but also increases the risk of cardiovascular diseases [7].

Despite the availability of various treatment options, including lifestyle interventions and pharmacotherapy that can effectively manage both diabetes and hyperlipidemia, these conditions are often addressed only after the development of hyperlipidemia in patients with diabetes [8]. This is largely due to the common practice of treating diabetes and hyperlipidemia separately rather than considering their coexistence and combined impact [9]. Consequently, treatment of diabetic with hyperlipidemia often begins only after its onset, limiting the effectiveness of interventions and increasing the risk of long-term complications. The 2018 ACC/AHA guidelines recommend moderate-intensity statin therapy for diabetic patients aged 40–75 years, with high-intensity statin therapy for high-risk patients or those with established atherosclerotic cardiovascular disease, aiming to keep LDL-C < 70 mg/dL; ezetimibe or a PCSK9 inhibitor may be added if this target is not reached [10]. This also suggests that preventive treatment of hyperlipidemia in diabetic patients is necessary in the early stages of the disease.

Currently, there is no reliable method to predict which T2D patients are at a risk of developing hyperlipidemia. Existing risk assessment tools primarily rely on established metabolic disorders rather than on predicting the potential risk of hyperlipidemia [11]. While certain gene polymorphisms have been associated with an increased risk of hyperlipidemia in patients with diabetes, the predictive capability and practical application of these genetic markers still require further validation [12]. Traditional risk factors, such as age, duration of diabetes, and obesity, although providing some insights, are insufficient to accurately predict the occurrence of hyperlipidemia in patients [13]. This highlights a significant gap in the current understanding and management of diabetes with hyperlipidemia, emphasizing the need for more advanced predictive tools and biomarkers to identify high-risk individuals before the onset of hyperlipidemia.

In recent years, researchers have begun to explore the role of Proprotein Convertase Subtilisin/Kexin Type 9 (PCSK9) in the development of diabetes with hyperlipidemia. PCSK9 is a protein that regulates the blood concentrations of low-density lipoprotein (LDL) cholesterol by facilitating the degradation of LDL receptors [14]. PCSK9 levels are significantly higher in T2D patients than in the general population, and these elevated levels are associated with poorer metabolic parameters (such as total cholesterol, LDL cholesterol, and triglycerides) and an increased cardiovascular risk [15]. These findings suggest that PCSK9 is essential in the pathogenesis of diabetes with hyperlipidemia and could serve as a target for early intervention [16]. Understanding the role of PCSK9 in this context could open new avenues for predicting and preventing hyperlipidemia in T2D patients, thereby improving clinical outcomes and reducing cardiovascular risk.

This study aimed to combine clinical and laboratory indicators with PCSK9 to develop a predictive model for clinical application, specifically to identify the early clinical risk factors for the development of hyperlipidemia in T2D patients. Using this model, the risk of early hyperlipidemia in patients with T2D can be identified, thereby providing a basis for early intervention and treatment.

## Materials and methods

### The study population

Patients with Type 2 diabetes (T2D) were collected from August 1, 2022, to February 1, 2024. The study population consisted of village-level community health residents from Yuhuan City and patients diagnosed with T2D at Yuhuan People’s Hospital, all of whom adhered to the established inclusion and exclusion criteria.

Inclusion Criteria: 1.Patients diagnosed with T2D should be based on at least one of the following criteria: Fasting plasma glucose (FPG) ≥ 7.0 mmol/L; Random plasma glucose (RPG) ≥ 11.1 mmol/L with symptoms of diabetes; 2-hour plasma glucose ≥ 11.1 mmol/L during an oral glucose tolerance test (OGTT); Hemoglobin A1c (HbA1c) ≥ 6.5%. 2.Patients were required to have had a stable course of treatment without any major changes. 3.Patients were required to have a diabetes duration of at least 6 months. 4.The patients’ Body Mass Index (BMI) should be between 18.5 and 35 kg/m^2^.

Exclusion Criteria: 1.Patients diagnosed with hyperlipidemia, including but not limited to the following conditions: Low-Density Lipoprotein Cholesterol (LDL-C) ≥  4.1 mmol/L; Total Cholesterol (TC) ≥  6.2 mmol/L; Triglyceride (TG) ≥  2.3 mmol/L. 2. Age < 18 years or > 80 years. 3. Type 1 diabetes mellitus patients. 4. Patients currently receiving statin therapy or other lipid-lowering medications. 5. Patients with severe liver or kidney dysfunction. 6. Patients with severe diabetic complications. 7. Patients with cardiovascular diseases. 8. Patients with confirmed severe cardiovascular disease. 9. History of malignancy or other major disease.

### Collection of clinical data

T2D patients undergo physical examinations every six months. If they met the criteria for hyperlipidemia, clinical data from the previous examination were collected through the electronic medical record system (HIS) of Yuhuan People’s Hospital. Data collection for this study spanned from August 1, 2022, to August 1, 2024. The demographic and disease-related information included sex, age, smoking history, and comorbidities. Laboratory test results were as follows: Lp(a), TG, TC, LDL-C, HDL-C, ApoB/ApoA1, FBG, HbA1c, hs-CRP, serum creatinine, serum uric acid, WBC, hemoglobin, serum albumin, ALT, AST, PCSK9, and disease duration. Patients were divided into a T2D group, a T2D with hyperlipidemia group, and a healthy control group.

### PCSK9 test

Whole blood from all subjects was placed in vacuum coagulation vessels, centrifuged at 1000 ×  g for 20 min, and serum was separated and stored at −80°C for later use. Serum PCSK9 content was determined using the human proprotein convertase subtilisin/kexin type 9 (PCSK9) enzyme-linked immunosorbent assay kit (JONLN Biotech, China). Absorbance values were measured using a Molecular Devices microplate reader. A standard curve was plotted to calculate sample concentration. The ratio of sample concentration to the maximum value in the linear range of the standard curve was used for subsequent statistical analyses.

### Ethical statement

The study protocol was approved by the Ethics Committee of Yuhuan People’s Hospital, and informed consent was obtained from all patients. All the procedures were conducted in accordance with the principles of the Declaration of Helsinki.

### Statistical analysis

Data analysis was performed using R statistical software (version R4.4.0). Continuous variables are presented as mean ±  standard deviation (x ± s) or median (P25, P75), depending on the normality of their distribution. For normally distributed data across the three groups, ANOVA was employed. The Kruskal-Wallis test was used to compare non-normally distributed data among the three groups. For comparisons between two groups with non-normal distributions, the Mann-Whitney U test was applied. Independent-sample t-tests were used for normally distributed data. Categorical and ordinal data were expressed as counts, percentages, or ratios, and group comparisons were conducted using the chi-square test. The “dplyr” and “corrplot” packages in R were used to create correlation matrices for clinical and laboratory indicators. The LASSO regression method from the “glmnet” package was used to select features influencing the STR. A nomogram model and calibration curves were constructed using the “rms” package, and the receiver operating characteristic (ROC) curve was plotted using the “pROC” package. Before conducting the LASSO regression analysis, the T2D and T2D with hyperlipidemia groups were randomly divided into training (70%) and test (30%) sets.

## Results

### Clinical characteristics and laboratory characteristics

**Fig 1:**
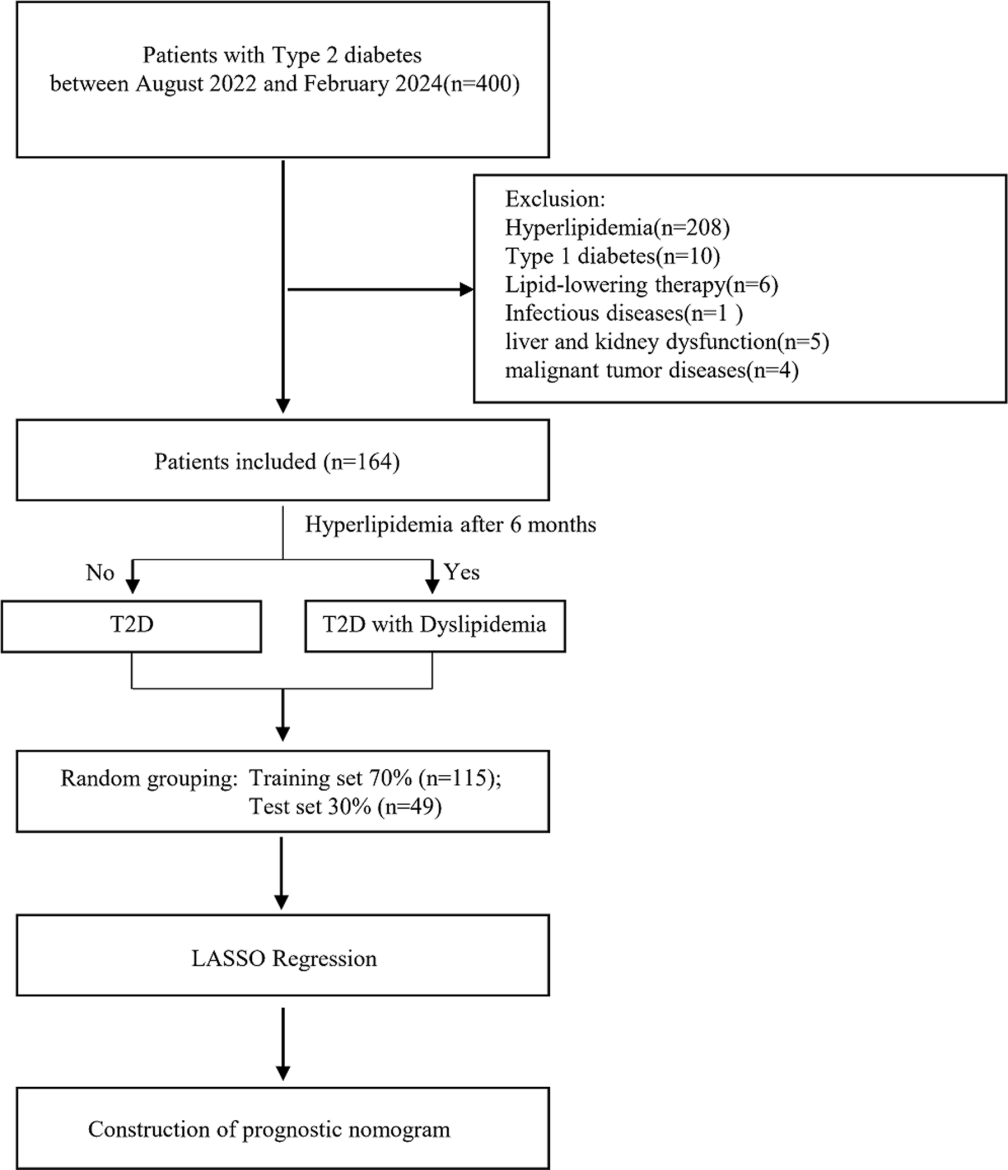
Study Flowchart. The diagram illustrates the methodology and participant selection criteria employed in the research.

**Fig 2:**
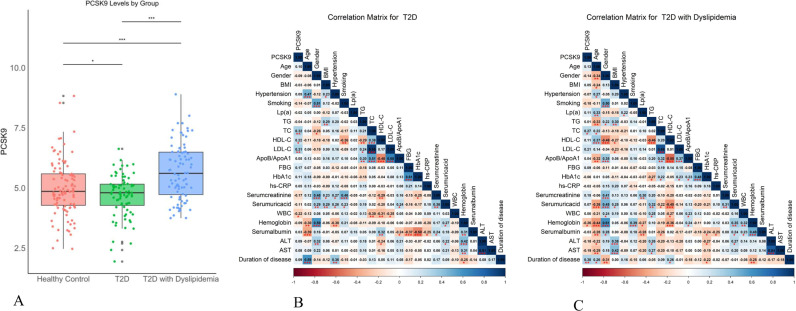
Laboratory Results and Correlation Analysis. (A) Scatter and box plots illustrate the levels of PCSK9 in different groups. (B) Correlation matrix for clinical and laboratory indices in T2D group. (C) Correlation matrix for the T2D with hyperlipidemia group. * P < 0.05, ***P < 0.001.

Fig 1 shows the flowchart of the study. The study included 105 healthy individuals, 80 T2D patients, and 84 patients with both T2D and hyperlipidemia. Compared with the healthy group, the BMI and hypertension rates were significantly higher in the diabetes and diabetes with hyperlipidemia groups, although no notable differences were found in age and gender (Table1 in S1 Table). The BMI of the diabetes with hyperlipidemia group was significantly higher than that of the diabetes group (Table 1). When the three groups were compared, significant differences were found in Lp(a), TG, TC, HDLC, LDL-C, ApoB/ApoA1, FBG, HbA1c, hs-CRP, serum creatinine, WBC, hemoglobin, ALT, and PCSK9 levels (Table1 in S1 Table). Compared with the T2D group, the T2D with hyperlipidemia group had significantly higher levels of TG, TC, LDL-C, ApoB/ ApoA1, FBG, HbA1c, hs-CRP, and PCSK9 (Fig 2A). No significant differences were found between the two cohorts in terms of Lp(a), HDLC, serum creatinine, serum uric acid, WBC, hemoglobin, serum albumin, ALT, AST, and disease duration (Table 1). Correlation matrix analysis was conducted for the clinical and laboratory parameters listed in Table 1. The numbers in the figure represent r-values with p-values indicated by asterisks. The correlation matrix revealed that in the T2D group, PCSK9 was positively correlated with TC, HDLC, and LDL-C and negatively correlated with WBC. In the T2D with hyperlipidemia group, PCSK9 exhibited a positive correlation with TC and disease duration, while demonstrating a negative correlation with hemoglobin (Fig 2B-C).

**Table 1:** Demographic and Clinical Characteristics of Patients.

Variable	Healthy Control n = 105	T2D n = 80	T2D with Dyslipidemia n = 84	P_value
Age	51 (40,61)	51 (45,59)	51 (45,58)	0.847
Gender	63 (60%)	51 (63.75%)	54 (64.29%)	1.000
BMI	23.5 (21.8,25.3)	23.8 (22.4,25.7)	24.9 (23.1,26.9)	0.018
Hypertension	11 (10.48%)	27 (33.75%)	31 (36.9%)	0.796
Smoking	30 (28.57%)	25 (31.25%)	26 (30.95%)	1.000
Lpa	94 (56,167)	132.5 (68.25,288.25)	134 (70,274)	0.993
TG	0.93 (0.77,1.16)	1.08 (0.81,1.34)	1.58 (0.93,2.16)	0.001
TC	4.46 (4.06,4.79)	4.22 (3.65,4.70)	5.30 (4.76,5.81)	0.001
HDLC	1.32 (1.14,1.44)	1.12 (0.97,1.34)	1.19 (1.03,1.29)	0.440
LDLC	2.84 (2.55,3.11)	2.79 (2.36,3.2)	3.54 (3.16,3.96)	0.001
ApoBA1	0.62 ± 0.16	0.72 ± 0.2	0.96 ± 0.24	0.001
FBG	4.94 (4.65,5.18)	6.85 (6.02,9.14)	9.14 (7.56,11.29)	0.001
HbA1c	5.5 (5.3,5.8)	8.3 (7.0,10.5)	10.3 (9.0,11.9)	0.001
hsCRP	0.7 (0.4,1.5)	0.8 (0.48,1.4)	1.3 (0.9,2.2)	0.001
Serum creatinine	69 (56,78)	59.5 (49,70)	60.5 (51,69.25)	0.878
Serum uric acid	318.55 ± 70.87	328.94 ± 78.55	317.04 ± 89.18	0.365
WBC	5.2 (4.2,5.9)	5.7 (4.97,7.1)	5.8 (5.07,6.8)	0.917
Hemoglobin	141 (128,148)	146 (133, 156)	146 (135,158)	0.753
Serum albumin	43.6 (42.1,45.1)	42.3 (39.6, 45.2)	42.2 (38.7,45.0)	0.613
ALT	16 (12,22)	21 (17,32)	26 (17.75,33)	0.257
AST	21 (18,25)	21 (16.75,25.25)	21 (16.75,28)	0.550
PCSK9	4.86 (4.28,5.59)	4.81 (4.28,5.16)	5.61 (4.73,6.49)	0.001
Duration of disease		48 (15,97.25)	29.5 (12,111.75)	0.642

BMI, Body Mass Index; TG, Triglycerides; TC, Total Cholesterol; FBG, Fibrinogen; ALT, Alanine Aminotransferase; AST, Aspartate Transaminase; PCSK9, Proprotein Convertase Subtilisin/Kexin Type 9. P-value: Comparison between the T2D group and the T2D with hyperlipidemia group.

### Risk factors for the development of hyperlipidemia in T2D patients

**Fig 3:**
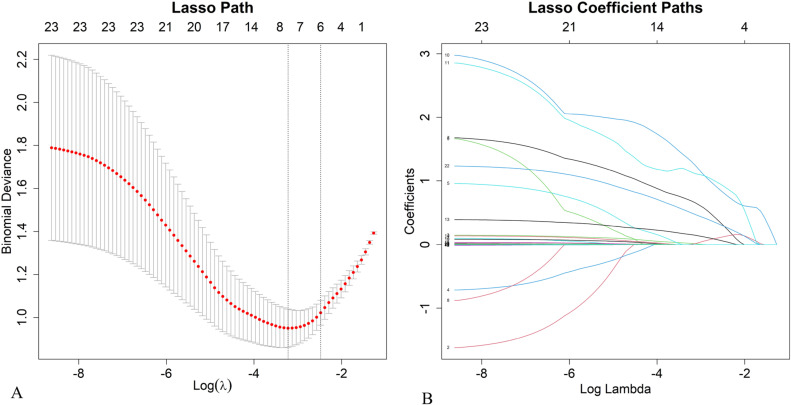
LASSO regression analysis of risk factors. (A) Coefficient paths for selected variables. (B) Cross-validation for optimal λ selection.

LASSO regression analysis was used to reduce the dimensionality of the 9 statistically significant factors (BMI, TG, TC, LDL-C, ApoB/ApoA1, FBG, HbA1c, hs-CRP, PCSK9) (Fig 3A-B). The optimal λ (best λ: 0.0398) value was determined by 10-fold cross-validation to minimize the cross-validation error, which helped further select the variables associated with diabetes combined with hyperlipidemia. Six variables with nonzero regression coefficients were identified: BMI, TG, TC, LDL-C, HbA1c, and PCSK9. These six variables were found to be predictors of diabetes and hyperlipidemia.

### Nomogram model construction

**Fig 4:**
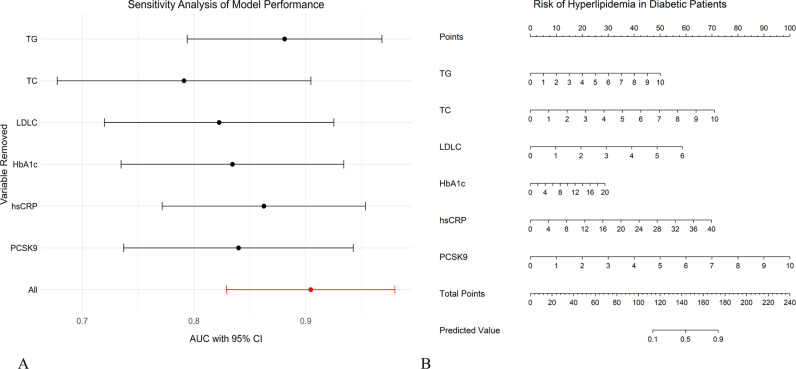
Sensitivity Analysis of the Nomogram. (A) Sensitivity analysis of model performance on the Area Under the Curve (AUC) with 95% Confidence Interval (CI). (B) BMI, TG, TC, LDL-C, HbA1c, and PCSK9 were selected to construct the nomogram model.

Multivariate logistic regression analysis was conducted based on the factors identified by LASSO regression (Table 2). Using the “rms” package in R, a prototype nomogram for predicting the occurrence of hyperlipidemia in T2D patients. The total score is computed by summation of the scores from the six indicators presented in Table 2. The risk of hyperlipidemia in diabetic patients is represented by the vertical line from the total score to the horizontal axis labeled “Risk of Hyperlipidemia in Diabetic Patients” (Fig 4B).

**Table 2:** The variables identified by LASSO regression.

Variable	Beta	SE	Wald Statistic	P-value	OR	95% CI Lower	95% CI Upper
TG	0.423	0.409	1.035	0.301	1.527	0.688	3.474
TC	0.680	0.307	2.215	0.027	1.974	1.103	3.735
LDL-C	0.935	0.460	2.033	0.042	2.547	1.062	6.566
HbA1c	0.111	0.079	1.400	0.161	1.117	0.957	1.309
ApoB/ApoA1	0.240	0.993	0.242	0.809	1.272	0.186	9.531
PCSK9	0.384	0.186	2.070	0.038	1.469	1.033	2.154
BMI	0.038	0.048	0.786	0.432	1.039	0.945	1.143

SE, Standard Error; OR, Odds Ratio; CI, Confidence Interval.

### Sensitivity analysis of the nomogram

**Fig 5:**
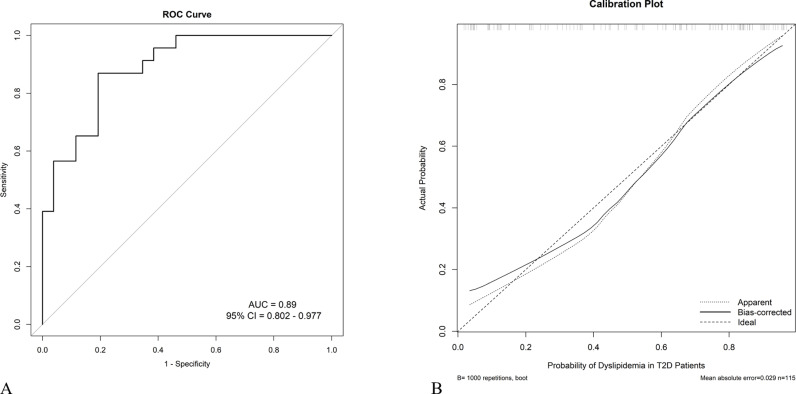
ROC Curves of the Nomogram. (A) ROC curve showing the nomogram’s predictive accuracy (AUC =  0.89, 95% CI: 0.802–0.977). (B) Calibration plot indicating good agreement between predicted and actual probabilities.

Considering the interactions between indicators, a sensitivity analysis was conducted by removing each of the five indicators (BMI, TG, TC, LDL-C, HbA1c, and PCSK9) individually. The results showed that the AUC 95% confidence interval (CI) for each removal was worse than that for all the indicators combined (Fig 4A). Therefore, BMI, TG, TC, LDL-C, HbA1c, and PCSK9 were selected to construct the nomogram model (Fig 4B), which had an AUC under 0.89 (95% CI 0.802–0.977) (Fig 5A). The internal calibration plot, drawn using bootstrapping, showed that the risk-fit curve of the nomogram closely matched the ideal curve, indicating a good calibration of the nomogram (Fig 5B).

### Clinical applicability analysis of the nomogram model

**Fig 6:**
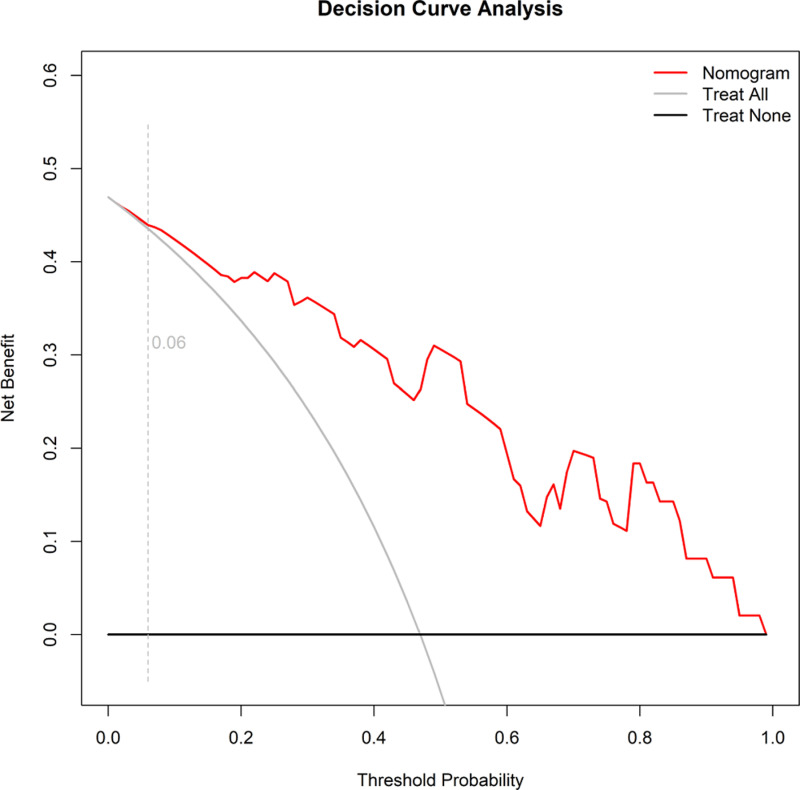
Decision Curve Analysis. Decision curve analysis of the nomogram, showing net benefit at varying threshold probabilities.

A clinical decision curve was constructed using nomogram prediction probability as the test variable and T2D with hyperlipidemia as the status variable. When the predicted probability of T2D with hyperlipidemia exceeds 0.06, the net benefit of applying the nomogram is higher than that of the “Treat None” and “Treat All” strategies, indicating that the nomogram has good clinical applicability (Fig 6).

## Discussion

T2D is a chronic metabolic disorder that is often accompanied by a range of complications, with hyperlipidemia being among the most common [17]. The prevalence of hyperlipidemia is significantly higher in patients with T2D than in the general population [18]. This condition is characterized by elevated levels of TG, TC, HDL-C, and LDL-C, which contribute to increased cardiovascular risk in patients with T2D [19]. Identifying and understanding the risk factors leading to hyperlipidemia in T2D patients is crucial for improving patient outcomes and guiding therapeutic interventions.

Currently, few predictive models are available to assess the risk of hyperlipidemia and its associated cardiovascular risk in T2D patients. These models typically combine clinical and biochemical parameters to estimate the likelihood of developing hyperlipidemia [20]. Traditional models often include factors such as age, gender, BMI, duration of diabetes, and glycemic control, as measured by HbA1c [21]. Machine learning methods, such as LASSO regression, have also been applied in clinical settings due to their advantages in model construction [22]. More advanced models have begun to incorporate genetic and molecular markers, acknowledging the complex interactions between genetics, metabolism, and lipid disorders [23,24]. However, despite these advancements, there remains a need for more precise and personalized models to accurately predict the risk of hyperlipidemia in the T2D population. The complexity of lipid metabolism and its regulation suggest that multifactorial models incorporating a broader range of clinical, biochemical, and molecular factors may offer improved predictive accuracy [25,26].

In this study, T2D patients were divided into two cohorts based on whether they developed hyperlipidemia after six months, and their clinical data and laboratory indicators were compared. Statistical analysis revealed significant differences between the two groups in BMI, TG, TC, LDL-C, ApoB/ApoA1, FGB, HbA1c, hs-CRP, and PCSK9 levels. LASSO regression analysis was used to screen for risk factors, leading to the construction of a nomogram model based on BMI, TG, TC, LDL-C, HbA1c, and PCSK9. This model demonstrated good predictive performance and clinical applicability, aiding in the early identification of the risk of hyperlipidemia in T2D patients.

PCSK9 is an emerging player in the field of lipid metabolism and is gaining attention for its critical role in regulating LDL-C levels [27]. PCSK9 directly influences the production of apolipoprotein B (apoB), which is crucial for the formation of triglyceride-rich lipoproteins such as very low-density lipoprotein (VLDL) and intermediate-density lipoprotein (IDL) [28]. Modulation of PCSK9 activity can improve lipid and glucose metabolism [29]. Previous studies have shown that PCSK9 concentrations are not only closely associated with LDL-C levels but also serve as a predictor of cardiovascular events in diabetic patients [30]. In this study, PCSK9 is identified as a valuable biomarker for predicting the risk of hyperlipidemia in T2D patients, consistent with previous findings. T2D patients with higher levels of PCSK9 in their blood are more likely to develop hyperlipidemia. The PCSK9 inhibitor evolocumab has been shown to significantly reduce LDL-C and other lipid markers, providing substantial therapeutic benefits for T2D patients with hyperlipidemia [31]. Furthermore, in T2D patients with hypercholesterolemia, PCSK9 levels may not be affected by ezetimibe or its combination with statins [32]. Therefore, PCSK9 may play a critical role in lipid management in T2D patients, particularly when traditional lipid-lowering therapies, such as statins, are ineffective or not well-tolerated [33]. However, studies suggest that in patients with poorly controlled T2D, PCSK9 does not significantly affect the catabolism of LDL-apoB100 [34]. Another study found no significant association between plasma PCSK9 levels and the incidence of new-onset diabetes [35]. Thus, controlling plasma PCSK9 levels in T2D patients may reduce the risk of hyperlipidemia and consequently lower cardiovascular risk.

Elevated TG levels are particularly common in T2D patients and are closely associated with insulin resistance and poor glycemic control [36,37]. TG-rich lipoproteins contribute to the formation of small, dense LDL particles, which are more atherogenic and increase cardiovascular risk [38]. TC and LDL-C are also key factors in the development of hyperlipidemia in T2D patients [39]. Elevated LDL-C is of particular concern because of its direct role in atherosclerosis [40]. In T2D patients, LDL particles tend to be smaller and denser, making them more likely to penetrate the arterial walls and promote plaque formation [41]. Therefore, controlling LDL-C levels is the primary target for the treatment of diabetes with hyperlipidemia. BMI is a well-known risk factor for hyperlipidemia [42]. In T2D patients, a higher BMI is often associated with insulin resistance, which exacerbates lipid abnormalities [43]. HbA1c, a marker of long-term glycemic control, is closely related with hyperlipidemia [44]. Poor glycemic control leads to the glycation of lipoproteins, altering their function and increasing atherogenic potential [45]. Additionally, elevated HbA1c levels are associated with increased TG and decreased HDL-C levels, further contributing to lipid imbalance in T2D [46].

In conclusion, the early identification and timely intervention of hyperlipidemia risk in T2D patients are crucial for improving cardiovascular health and reducing the incidence of associated complications and mortality. These measures will not only enhance patient quality of life but also alleviate the burden on healthcare systems. Despite the statistical significance of our findings, it is important to note that the small sample size may limit the reliability of the predictive model for large-scale clinical practice. In future research, we plan to increase the sample size and conduct more in-depth investigations into potential clinical risk factors, along with comprehensive data analysis and validation, to identify the key factors associated with the occurrence of hyperlipidemia in T2D patients. Through these efforts, our goal is to continuously optimize and refine the existing predictive models to make them more accurate and comprehensive. This will not only improve the risk assessment of hyperlipidemia in T2D patients but also provide clinicians with more detailed and personalized diagnostic and therapeutic recommendations.

## Supporting Information

S1 Table Comparison of clinical and laboratory parameters among Healthy, T2D, and T2D with Dyslipidemia groups. (DOCX)
