# Supplementary material for: A predictive model to assess the risk of developing hyperlipidemia in patients with type 2 diabetes
Source: PLoS One. 2025 Feb 14;20(2):e0315781. doi: 10.1371/journal.pone.0315781 (PMC11828393; doi:10.1371/journal.pone.0315781)
Supplement: S1 Table — (DOCX) [file pone.0315781.s001.docx]

**Table 1. Comparison of clinical and laboratory parameters among Healthy, T2D, and T2D with Dyslipidemia groups.**

| Variable | Healthy VS  T2D | Healthy VS T2D&Dyslipidemia | T2D VS T2D&Dyslipidemia | Healthy VS T2D VS T2D&Dyslipidemia |
| --- | --- | --- | --- | --- |
| Age | 0.359 | 0.848 | 0.847 | 0.433 |
| Gender | 0.912 | 1.000 | 1.000 | 0.966 |
| BMI | 0.205 | 0.001 | 0.018 | 0.001 |
| Hypertension | 0.001 | 0.001 | 0.796 | 0.001 |
| Smoking | 1.000 | 0.692 | 1.000 | 0.853 |
| Lpa | 0.010 | 0.047 | 0.993 | 0.026 |
| TG | 0.302 | 0.001 | 0.001 | 0.001 |
| TC | 0.028 | 0.001 | 0.001 | 0.001 |
| HDLC | 0.001 | 0.001 | 0.440 | 0.001 |
| LDLC | 0.372 | 0.001 | 0.001 | 0.001 |
| ApoBA1 | 0.001 | 0.001 | 0.001 | 0.001 |
| FBG | 0.001 | 0.001 | 0.001 | 0.001 |
| HbA1c | 0.001 | 0.001 | 0.001 | 0.001 |
| hsCRP | 0.468 | 0.001 | 0.001 | 0.001 |
| Serum creatinine | 0.001 | 0.001 | 0.878 | 0.001 |
| Serum uric acid | 0.342 | 0.610 | 0.365 | 0.697 |
| WBC | 0.001 | 0.001 | 0.917 | 0.001 |
| Hemoglobin | 0.001 | 0.001 | 0.753 | 0.001 |
| Serum albumin | 0.067 | 0.189 | 0.613 | 0.183 |
| ALT | 0.001 | 0.001 | 0.257 | 0.001 |
| AST | 0.973 | 0.089 | 0.550 | 0.197 |
| PCSK9 | 0.302 | 0.001 | 0.001 | 0.001 |
| Duration of disease |  |  | 0.642 |  |

BMI, Body Mass Index; TG, Triglycerides; TC, Total Cholesterol; FBG, Fibrinogen; ALT, Alanine Aminotransferase; AST, Aspartate Transaminase; PCSK9, Proprotein Convertase Subtilisin/Kexin Type 9. P-values were calculated using ANOVA, Kruskal-Wallis, chi-square, t-test, or Mann-Whitney U test, as appropriate.
